# Effects of Backward Walking on External Knee Adduction Moment and Knee Adduction Angular Impulse in Individuals with Medial Knee Osteoarthritis

**DOI:** 10.3390/bioengineering12101057

**Published:** 2025-09-29

**Authors:** Min Zhang, Sizhong Wang, Jiehang Lu, Jian Pang, Peige Wang, Bo Chen, Hongsheng Zhan

**Affiliations:** 1 Department of Orthopedics & Traumatology, Shuguang Hospital, Shanghai University of Traditional Chinese Medicine, No. 528 Zhangheng Road, Pudong New Area, Shanghai 201203, China; 13761730320@163.com (J.L.); pangjian@shutcm.edu.cn (J.P.); peige_wang@163.com (P.W.); cbm818@shutcm.edu.cn (B.C.); zhanhongsheng@shutcm.edu.cn (H.Z.); 2 School of Health Sciences, University of Salford, Manchester M6 6PU, UK; 3 Division of Physiotherapy, Department of Health Sciences, College of Health, Medine and Life Sciences, Brunel University London, London UB8 3PH, UK; sizhong.wang@brunel.ac.uk; 4 Centre for Physical Activity in Health and Disease (CPAHD), Brunel University London, London UB8 3PH, UK

**Keywords:** backward walking, knee osteoarthritis, knee loading, gait analysis

## Abstract

**Background:** Backward walking (BW) has been proven to reduce the external knee adduction moment (EKAM) and knee adduction angular impulse (KAAI) during gait in healthy subjects, but its effects in individuals with knee osteoarthritis (OA) remain unknown. This study aimed to investigate the effects of self-selected speed BW on the EKAM, KAAI, and external knee flexion moment (EKFM) in individuals with medial knee OA. **Methods:** Thirty-two participants with medial knee OA underwent a three-dimensional gait analysis across three randomized conditions: (1) self-selected speed forward walking (FW), (2) self-selected speed BW, and (3) speed-controlled forward walking (SCFW) (for each individual, the SCFW speed was controlled within a range of 95% to 105% of BW speed). For each condition, the first peak of EKAM, second peak of EKAM, first peak of EKFM, and the KAAI were determined. One-way repeated measures ANOVA and multiple pairwise comparisons were performed to compare peaks of EKAM, peak of EKFM, and the KAAI between conditions. **Results:** BW significantly reduced the first peak of EKAM and the KAAI in comparison with FW and SCFW (*p* < 0.001). Both BW and SCFW showed a significantly reduced first peak of EKFM in comparison with FW (*p* < 0.001). However, BW did not reduce the second peak of EKAM when compared with FW or SCFW (*p* > 0.05). **Conclusions:** BW can significantly reduce the first peak of EKAM and the KAAI in comparison with FW and SCFW in individuals with medial knee OA.

## 1. Introduction

Knee osteoarthritis (OA) is one of the most common musculoskeletal disorders that cause disability and chronic pain in people aged 60 years or above [1]. The latest systematic review estimated that the prevalence of symptomatic knee OA in mainland China is 14.6% [2]. The common symptoms of knee OA are knee joint pain, stiffness, and loss of joint range of motion (ROM), which can seriously limit patients’ daily physical and social activities, and result in poor quality of life [3].

Excessive knee loading has been proven to be positively correlated with pain caused by knee OA [4,5]. However, direct measurement of loading at the knee during daily activity is not possible. Hence, the external knee adduction moment (EKAM), which is mainly determined by the ground reaction force (GRF), and the knee moment arm in the frontal plane have been widely accepted as a surrogate measure of the medial contact force during gait. The EKAM is a significant predictor of medial knee contact force, accounting for approximately 63% of the variance [6,7,8,9]. A previous study reported that every one-unit increase in the EKAM (Nm/BW·Ht%) is associated with more than a sixfold increase in the risk of medial knee OA progression [10]. Moreover, an increase in the EKAM is associated with a decrease in the knee joint space width [11]. Therefore, many treatment approaches, such as lateral wedges and gait retraining modifications, focus on reducing the peak EKAM during walking as their primary target [12,13].

Although the EKAM has been shown to be the best surrogate measure of medial contact force, a decreased EKAM cannot guarantee a decreased medial contact force during gait [14]. Walter’s study [14] demonstrated that a reduction in the first peak of EKAM did not correspond to a reduction in the first peak of medial contact force. In contrast, a reduction in the knee adduction angular impulse (KAAI) corresponded to reductions in impulse of medial contact force. Furthermore, the peaks of medial contact force were best predicted by a combination of the EKAM peaks and the peak of the external knee flexion moment (EKFM), as the peak values of EKFM have been shown to account for approximately 22% of the variance in the peak medial contact force [9]. Therefore, a comprehensive assessment of medial knee loading during gait should incorporate both the EKFM and KAAI, along with the EKAM, to better capture the biomechanical factors influencing the contact force [15,16].

The effectiveness of backward walking (BW) in the management of knee OA has been investigated by some previous studies [17,18,19,20]. A recent meta-analysis further supported BW as an effective supervised rehabilitation strategy for managing knee OA [18]. Several studies have reported that short-to-medium-term BW can help reduce pain, enhance knee joint stability, and improve physical activity in individuals with knee OA [17,19,20]. Alghadir et al. (2019) [17] demonstrated that a 6-week BW program significantly improved pain levels, physical function, and quadriceps strength. Similarly, Gondhalekar and Deo (2013) [19] found that a 3-week BW intervention significantly reduced knee pain and enhanced knee joint range of motion (ROM), as well as hip abductor and extensor strength. Goonasegaran et al. (2022) [20] also reported significant improvements in pain and physical function following a 12-week BW training program.

Although the clinical effectiveness of BW on knee OA has been reported, it remains uncertain whether the reduction in pain following BW training is associated with alterations in the EKAM and KAAI. One previous study indicated that BW significantly reduced the first and second peaks of EKAM and KAAI despite the increased first peak of vertical GRF in healthy individuals, which indicated that it could be a potential strategy for reducing the medial contact force [21]. Therefore, the potential mechanism of BW for improving pain might be correlated with the reduction in the first peak of EKAM, as it has been proven to be positively associated with pain in individuals with knee OA [16,22]. However, it remains unclear whether BW can reduce the first peak of EKAM, second peak of EKAM, and the KAAI in individuals with medial knee OA. In addition, the walking speed during BW is generally slower in comparison with FW, due to a person not being able to see the direction of progress [21,23], and whether the reduction in peaks of EKAM and the KAAI during BW is due to the reduction in walking speed is still unclear [23,24].

Therefore, this study aimed to investigate the immediate biomechanical effects of BW on first and second peaks of EKAM, peak of EKFM, and the KAAI in individuals with medial knee OA. Based on the findings of a previous study that BW significantly reduced the first and second peaks of EKAM and the KAAI in comparison with FW in healthy participants [21], we hypothesized that the first and second peaks of EKAM and the KAAI would be reduced by BW when compared with FW and speed-controlled FW (SCFW). We also hypothesized that the peak of EKFM would not change, as BW does not target a reduction in the EKFM.

## 2. Methods

### 2.1. Participants

Participants diagnosed with medial knee OA were recruited from outpatients of the Department of Orthopaedics & Traumatology of Shuguang Hospital by way of advertisements. All participants were screened by an experienced orthopedist at Shuguang Hospital. Participants were included if they (1) had radiographic medial tibiofemoral knee OA (defined as Kellgren & Lawrence grade I, II, or III) [25], and (2) were aged 40 years and above and could walk without any aid. Participants with any of the following conditions were excluded: (1) known learning disability, (2) any other musculoskeletal diseases that influenced their gait pattern, (3) a history of lower limb surgery, and (4) received treatment (i.e., acupuncture, massage, or physiotherapy) for the affected knees in the last four weeks. Individuals who met the inclusion criteria were provided with a detailed explanation of the study, along with a participant information sheet and a health history questionnaire. Those who agreed to participate and met the eligibility requirements were scheduled for an appointment at the gait laboratory within fourteen days. Upon arrival, any questions they had were answered, and they signed an informed consent form.

The sample size calculation was based on a previous similar study that explored the effects of BW on the EKAM in healthy individuals [21]; the effect size of BW on the 1st peak of EKAM in healthy individuals was 0.76 when compared with FW. However, given the potential discrepancy in the effect of BW on the 1st peak of EKAM between individuals with and without knee OA, we opted for a conservative approach and expected a smaller effect size of 0.6 (which is 20% less than the effect size reported by one previous study [21]) for individuals with knee OA, and we used an F-test (fixed-effects, omnibus, one-way ANOVA) with a sample power of 80% and an alpha value of 0.05. The analysis showed that a sample size of at least 30 participants would be adequate to power this study. We calculated the sample size using G*Power (Version 3.1.9.6, University of Kiel, Kiel, Germany) [26].

### 2.2. Ethics Statement

The study was conducted in accordance with the Declaration of Helsinki and approved by the China Ethics Committee of Registering Clinical Trials (ChiECRCT-20171564).

### 2.3. Data Collection

**Figure 1:**
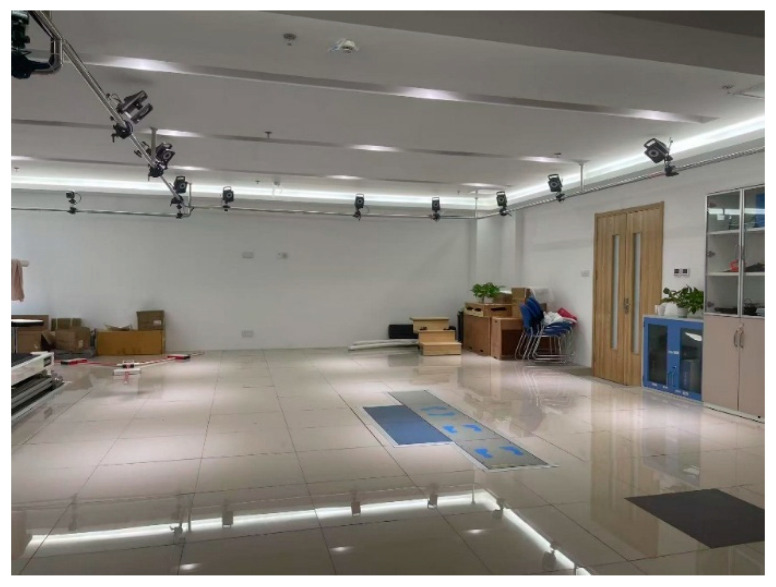
Sixteen-camera Vicon system and four AMTI force plates in the gait laboratory.

The kinematic data were collected using a 16-camera VICON motion capture system (Version 1.8.5, VICON, Oxford, UK) at 100 Hz. The GRF data were collected using two 400 × 600 mm^2^ AMTI force plates (OR6-6, AMTI, Watertown, MA, USA) at 1000 Hz, which were integrated and synchronized with the VICON system (Figure 1). Reflective markers were firmly affixed onto specific bony landmarks based on a previous study [27]. Standard shoes (Huili, Shanghai Huili Footwear Co., Ltd., Shanghai, China) offered by the investigators were used to minimize the possibility of interaction between shoes and the ground.

Before collecting the data, all participants had five trials of FW and BW at their comfortable pace, which were averaged to identify their self-selected speed in each condition. A Smartspeed timing gate system (Smartspeed PT, SMARTBASE, Brisbane, QLD, Australia) was used to monitor the speed in the SCFW condition. For each participant, the SCFW speed was similar to the BW speed, and the SCFW speed was controlled within a range of 95% to 105% of BW speed. Therefore, the SCFW speed was controlled to allow for a comparison between the BW and SCFW conditions.

Upon arrival at the gait laboratory, the participants were instructed to change into shorts and a comfortable T-shirt. A static trial was then conducted to calibrate the gait model prior to the gait tasks. All the FW, BW, and SCFW trials were performed in one session. The participants were asked to perform five trials in each of the three conditions in a randomized order. The sequence of FW, BW, and SCFW was randomly decided by asking the participants to draw a card from a box; each card had a different sequence. A period of 15 min between conditions was given to allow the participants to have a rest to minimize the fatigue effect. Considering the difficulties of BW and the possible fatigue effect on the biomechanical outcomes, the participants were allowed to have a 20 s short break between trials. The kinematic and kinetic data were presented in a stance phase that was normalized to 100%. The data normalization has been added to the data results and analysis. The GRF was normalized to body weight, and the EKAM was normalized to the participants’ body mass.

### 2.4. Data Processing

Visual 3D (Version 6.01.16, C-motion, Rockville, MD, USA) was used to calculate the kinematic and kinetic outcome measures. The kinematic data and analog data were filtered using a Butterworth 4^th^-order digital filter with a cut-off frequency of 6 Hz for the kinematics and 25 Hz for the analog data [28]. An inverse dynamics algorithm was used to calculate the primary biomechanical outcomes during stance phase for the trials conducted under FW, BW, and SCFW conditions. The biomechanical variables of interest in this study are defined in Table 1.

### 2.5. Statistical Analyses

The data from the affected knees were analyzed. The normality of selected parameter was assessed by Shapiro–Wilk tests. A one-way repeated measures ANOVA was used to examine the difference in the EKAM peaks, EKAM arms, KAAI, 1st peak of EKFM, and GRF peaks between conditions. If the *p*-value was significant (*p* ≤ 0.05), the test was followed by multiple pairwise comparisons with a Bonferroni correction. The adjustment for multiple comparisons was applied to the 1st peak of EKAM, the 2nd peak of EKAM, and the KAAI. Therefore, the significance level for the post hoc tests was set at 0.05/3. All the statistical analyses were performed using IBM SPSS Statistics for Windows, Version 16.0 (SPSS Inc., Chicago, IL, USA).

## 3. Results

A total of thirty-two individuals with medial knee OA were recruited and successfully went through the study. Their demographic and characteristic data are summarized in Table 2. The Shapiro–Wilk tests showed that both the kinematic and kinetic parameters were normally distributed (*p* > 0.05). The biomechanical data for each condition are shown in Table 3. A significant reduction in walking speed was observed during BW when compared with FW (*p* < 0.001, mean difference = −0.26, 95% CI (−0.32 to −0.18)). No significant difference in walking speed was found between the BW and SCFW conditions (*p* > 0.05).

**Figure 2:**
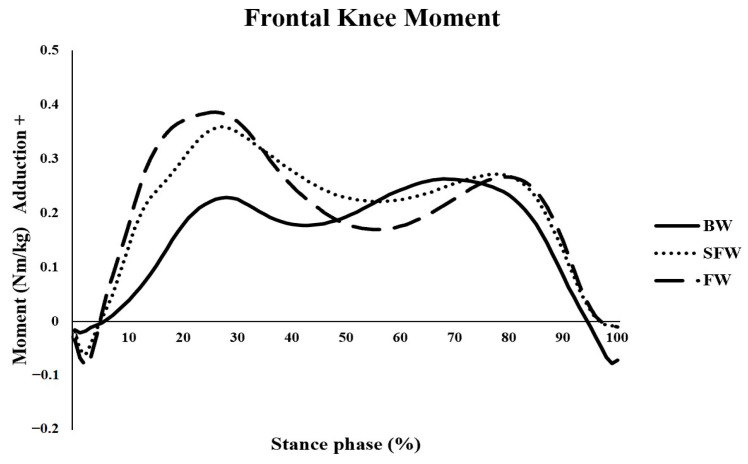
External knee adduction moment (EKAM) during backward walking (BW), speed-controlled forward walking (SCFW), and forward walking (FW).

BW significantly reduced first peak of EKAM in comparison with FW (*p* < 0.001, mean difference = −0.13, 95% CI (−0.18 to −0.08)) and SCFW (*p* < 0.001, mean difference = −0.10, 95% CI (−0.15 to −0.05)) (Figure 2). The first peak EKAM arm during BW was significantly reduced in comparison with FW (*p* < 0.001, mean difference = −0.02, 95% CI (−0.03 to −0.02)) and SCFW (*p* < 0.001, mean difference = −0.02, 95% CI (−0.03 to −0.01)). Furthermore, BW significantly reduced the KAAI in comparison with FW (*p* < 0.001, mean difference = −0.02, 95% CI (−0.04 to 0.01)) and SCFW (*p* < 0.001, mean difference = −0.06, 95% CI (−0.08 to −0.03)). No significant difference in the second peak of EKAM and the second peak EKAM arm between conditions was found (*p* > 0.05). BW showed a significantly reduced first peak of EKFM in comparison with FW (*p* < 0.001, mean difference = −0.07, 95% CI (−0.15 to 0.01)). However, the first peak of EKFM during BW was significantly increased in comparison with SCFW (*p* < 0.001, mean difference = 0.13, 95% CI (0.05 to 0.21)) (Table 3).

The first peak of vertical GRF during BW was significantly higher than that during FW (*p* < 0.001, mean difference = 0.07, 95% CI (0.03 to 0.11)) and SCFW (*p* < 0.001, mean difference = 0.14, 95% CI (0.10 to 0.17)) conditions (Table 3). However, the second peak of vertical GRF during BW was significantly reduced in comparison with that during FW (*p* < 0.001, mean difference = −0.10, 95% CI (−0.13 to −0.06)) and SCFW (*p* < 0.001, mean difference = −0.12, 95% CI (−0.15 to −0.08)). Both the early-stance medial–lateral GRF and late-stance medial–lateral GRF were found to act in the opposite direction under the BW condition. For FW and SCFW, the early-stance medial–lateral GRF and late-stance medial–lateral GRF were positive and the GRF acted medially, while it acted laterally (negative) during BW (Table 2). 

## 4. Discussion

The aim of this study was to examine the effects of BW on the EKAM, KAAI, and EKFM in comparison with natural speed FW and SCFW (similar speed with BW) in individuals with medial knee OA. Our results show that BW significantly reduced the first peak of EKAM as well as the KAAI in comparison with FW and SCFW. Such observations are supported by the reduction in first peak of EKAM arm during BW when compared with FW and SCFW during gait.

In comparison with FW, BW significantly reduced the first peak of EKAM by 33.3% in individuals with medial knee OA. This finding is in accordance with a previous study, which demonstrated a 26.3% reduction in the first peak of EKAM by BW during gait in healthy individuals [21]. The 33.3% reduction in first peak of EKAM achieved during BW is clinically relevant for knee OA, as the risk of progression of medial knee OA has been proven to be sensitive to changes in the first peak of EKAM [10]. The 33.3% reduction in EKAM suggests that BW may have the potential to reduce the risk of knee OA progression, with an effect greater than that of some non-surgical interventions, such as lateral wedges (9.3% reduction) [29] and gait retraining modification (20% reduction) [30]. It is generally accepted that the EKAM is positively correlated with walking speed, as an increased walking speed leads to a greater GRF and then EKAM [31,32]. Compared with FW, we found that BW decreased walking speed; however, the first peak of vertical GRF during BW was significantly greater than that during FW, which indicates that the reduction in the EKAM could be explained by the reduction in the moment arm rather than the decreased GRF. Compared with SCFW, BW showed a significant reduction in the first peak of EKAM of 27.8%. Since there was no difference in the walking speed between SCFW and BW, and BW also showed a significantly greater first peak of vertical GRF, the reduction in the EKAM could also be explained by the shorting of moment arm caused by the opposite direction of medial–lateral GRF during BW. One previous study indicated that the reduction in the EKAM arm during BW is caused by the opposite direction of medial–lateral GRF in the early stance during BW when compared with FW [21]. In our study, both FW and SCFW showed a positive medial–lateral (medial direction) GRF in early and late stances, whereas BW showed a negative medial–lateral GRF (lateral direction). Therefore, the reduction in the first peak of EKAM during BW was caused by the change in direction in the medial–lateral GRF.

The effects of BW on the second peak of EKAM during gait were inconsistent. One previous study showed that BW significantly reduced the second peak of EKAM [21]. However, we found that BW did not change the second peak of EKAM in comparison with FW. These inconsistent findings could be explained by the differences between the first and second peaks of vertical GRF, as previous studies have shown that individuals with knee OA have a greater second peak of vertical GRF than first peak of vertical GRF due to abnormal loading during gait [33,34].

Both BW and SCFW significantly reduced the first peak of EKAM compared with FW. However, SCFW exhibited a greater KAAI in comparison with FW and BW. Admittedly, a reduced walking speed can help to decrease the peak of EKAM during stance due to the reduction in the GRF [31]. However, the KAAI has been reported to be more sensitive to changes in walking speed when compared with first peak of EKAM [31], as a previous study demonstrated that a decrease in walking speed of 14% resulted in an increase in the KAAI of 12%, with no significant difference in the first peak of EKAM. In our study, SCFW showed a decrease in walking speed of 23%, which led to an increase in KAAI of 23%. The increased KAAI indicates that the knee was exposed to a longer duration of medial loading, which might accelerate the development and progression of medial knee OA [15]. Therefore, we should reconsider whether a reduced walking speed is good for reducing the loading at the knee during gait, even though one previous study recommended it as a potential strategy for individuals with medial knee OA [35].

Our findings show that BW significantly reduced the first peak of EKFM when compared with FW, which is supported by the findings from previous studies [23,36]. This findings can be explained by the difference in foot position at initial contact between the conditions. During FW, the vector of GRF is anterior to the knee at initial contact and then moves posterior to the knee [37]. However, during BW, the foot displays toe striking at the initial contact rather than heel striking, and the vector of GRF is positioned at the posterior of the knee later; therefore, BW showed a significantly reduced peak of EKFM in comparison with FW. One previous study [14] demonstrated that reducing the EKAM while simultaneously increasing the EKFM may result in no net change in the medial knee contact force. In contrast, our findings show that BW effectively reduced the first peak of EKAM without increasing the EKFM. Therefore, BW may serve as a more effective gait modification strategy for reducing the medial knee contact force and potentially slowing the progression of medial knee OA.

Our study demonstrates that BW effectively reduced the first peak of EKAM, the KAAI, and first peak of EKFM in comparison with FW. Given that the peaks of EKAM, KAAI, and EKFM have been reported to be positively correlated with the pain symptoms of medial knee OA [10,38,39,40], the reduction in pain by BW in individuals with knee OA might be correlated with the reduction in peak values of EKAM. Although BW was found to be safe and effective for improving pain, physical function, and stability of knee OA in a previous study [20], it is important for patients to maintain their focus during BW practice to reduce the risk of falling, as they cannot see the direction of progress [41].

The findings of the present study demonstrate the immediate effects of BW on medial knee loading in individuals with knee OA, suggesting its potential role in reducing the risk of disease progression. Previous studies have reported that 3- to 12-week BW interventions can alleviate pain in individuals with knee OA [17,19,20]. However, it remains unclear whether the observed pain reduction following BW training is directly associated with decreased knee loading. Furthermore, there is a lack of long-term follow-up studies investigating the effects of BW on pain, peak of EKAM, and the KAAI. Therefore, future studies should investigate the long-term clinical and biomechanical effects of BW in the management of knee OA, which may provide valuable insights into its underlying mechanisms.

There are several limitations of our study. Firstly, we only compared the effects of SCFW and FW with BW on the first peak of EKAM, second peak of EKAM, EKFM, and KAAI, which leaves the effects of BW at fast speed on these variables is still unclear. Secondly, we did not collect surface electromyography (sEMG) data for the lower limbs to identify muscle co-contraction during BW, and some previous studies have shown that muscle co-contraction can also influence knee loading [42,43]. Thirdly, we performed multiple tests in the current study, which could have increased the likelihood of type I error. Given that no previous studies have investigated the effects of BW on the EKAM, KAAI, and EKFM in individuals with medial knee OA, we did not define a primary outcome for the current study. Finally, we only investigated the immediate effects of BW on the first peak of EKAM, second peak of EKAM, EKFM, and KAAI during gait. The long-term effects of BW on pain, first peak of EKAM, second peak of EKAM, EKFM, and KAAI in individuals with medial knee OA are still unknown. Finally, the number of female participants with knee OA in this study was significantly higher than that of males, which may have potentially biased the results, as previous studies have shown that females with knee OA exhibit greater mediolateral gait regularity, higher stride frequency, and smaller EKAM compared with males [44,45].

## 5. Conclusions

This study confirms the immediate effects of BW for reducing the first peak of EKAM, KAAI, and the first peak of EKFM, which can be attributed to a decrease in the EKAM arm. However, it should be noted that this study was conducted under controlled conditions during a single session. Therefore, future studies are needed to better understand the potential benefits of long-term BW on medial knee loading in individuals with knee OA.

## Figures and Tables

**Table 1:** Biomechanical variables of interest.

Variable	Definition
1st EKAM (Nm/kg)	The peak of EKAM in the 1st half of the stance (from 1 to 33%).
2nd EKAM (Nm/kg)	The peak of EKAM in the 2nd half of the stance (from 68 to 100%).
1st EKAM arm (m)	The perpendicular distance between the GRF and knee joint center in the laboratory frontal plane, calculated at the time of 1st peak of EKAM.
2nd EKAM arm (m)	The perpendicular distance between the GRF and knee joint center in the laboratory frontal plane, calculated at the time of 2nd peak of EKAM.
KAAI (Nm/kg) · s	The positive area under the EKAM–time graph.
1st EKFM (Nm/kg)	The peak of EKFM in the 1st half of the stance (from 0 to 50%).
1st GRFz (body weight)	The peak of vertical GRF in the 1st half of the stance (from 0 to 50%).
2nd GRFz (body weight)	The peak of vertical GRF in the 2nd half of the stance (from 51 to 100%).
ESGRFx (body weight)	The peak of medial GRF in the 1st half of the stance (from 0 to 50%).
LSGRFx (body weight)	The peak of medial GRF in the 2nd half of the stance (from 51 to 100%).

1st EKAM = 1st peak of external knee adduction moment; 2nd EKAM = 2nd peak of external knee adduction moment; 1st EKAM arm = 1st peak of external knee adduction moment arm; 2nd EKAM arm = 2nd peak of external knee adduction moment arm; KAAI = knee adduction moment impulse; 1st EKFM = 1st peak of external peak knee flexion moment; 1st GRFz = 1st peak of vertical ground reaction force; 2nd GRFz = 2nd peak of vertical ground reaction force; ESGRFx = early-stance medial–lateral ground reaction force; LSGRFx = late-stance medial–lateral ground reaction force.

**Table 2:** Characteristics of participants (n = 32).

Variable	
Gender (male/female)	3/29
Age (years)	60.56 ± 4.93
Height (m)	1.59 ± 0.06
Body mass (kg)	58.20 ± 5.73
Body mass index (kg/m^2^)	23.17 ± 2.41
Kellgren & Lawrence grade of knee OA	grade 1 = 12, grade 2 = 15, grade 3 = 5

Values are mean ± SD unless otherwise indicated.

**Table 3:** Comparison of biomechanical data for BW, FW, and SCFW (bold indicates significance).

Variable	BW	FW	SCFW	*p* Value		
				Main Effect	BW vs. FW	BW vs. SCFW
Walking speed (m/s)	0.87 ± 0.12	1.12 ± 0.12	0.86 ± 0.11	**<0.001**	**<0.001**	1.000
1st EKAM (Nm/kg)	0.26 ± 0.12	0.39 ± 0.09	0.36 ± 0.09	**<0.001**	**<0.001**	**<0.001**
2nd EKAM (Nm/kg)	0.28 ± 0.09	0.27 ± 0.10	0.28 ± 0.11	0.785	0.700	1.000
1st EKAM arm (m)	0.04 ± 0.02	0.07 ± 0.02	0.06 ± 0.02	**<0.001**	**<0.001**	**<0.001**
2nd EKAM arm (m)	0.05 ± 0.02	0.05 ± 0.02	0.05 ± 0.02	0.446	0.179	0.166
KAAI (Nm/kg) · s	0.12 ± 0.05	0.13 ± 0.05	0.16 ± 0.05	**<0.001**	**<0.001**	**<0.001**
1st EKFM (Nm/kg)	0.31 ± 0.17	0.38 ± 0.18	0.18 ± 0.15	**<0.001**	**<0.001**	**<0.001**
1st GRFz (body weight)	1.13 ± 0.10	1.06 ± 0.09	1.00 ± 0.06	**<0.001**	**<0.001**	**<0.001**
2nd GRFz (body weight)	0.98 ± 0.08	1.09 ± 0.07	1.08 ± 0.06	**<0.001**	**<0.001**	**<0.001**
ESGRFx (body weight)	−0.08 ± 0.02	0.06 ± 0.01	0.05 ± 0.01	**<0.001**	**<0.001**	**<0.001**
LSGRFx (body weight)	−0.06 ± 0.01	0.05 ± 0.02	0.05 ± 0.02	**<0.001**	**<0.001**	**<0.001**

Values are the mean ± SD. FW = forward walking; BW = backward walking; SCFW = speed-controlled forward walking; bold = significant.

## Data Availability

The data presented in this study are available on request from the corresponding author due to ethical reasons.
